# Geniposide alleviates CCl_4_-induced liver fibrosis: Potential involvement of the PERK-CHOP signaling pathway

**DOI:** 10.3892/br.2026.2209

**Published:** 2026-09-30

**Authors:** Yue Yu, Xinyi Kwan, Huiqing Liang, Shaodong Chen

**Affiliations:** 1 Dongzhimen Hospital, Beijing University of Chinese Medicine, Beijing 100700, P.R. China; 2 Department of Traditional Chinese Medicine, School of Medicine, Xiamen University, Xiamen, Fujian 361102, P.R. China; 3 Center for Liver Disease, Xiamen Hospital of Traditional Chinese Medicine, Xiamen, Fujian 361000, P.R. China; 4 School of Traditional Chinese Medicine, Xiamen University Malaysia, Sepang, Selangor 43900, Malaysia

**Keywords:** geniposide, liver fibrosis, ER stress, apoptosis, CHOP, CCl_4._

## Abstract

Geniposide is the primary active component of the cycloether terpenoid glycoside class extracted from the traditional Chinese medicinal herb *Gardenia jasminoides*, and it possesses multiple pharmacological activities, including neuroprotection, antidiabetic effects and hepatoprotection. It has been shown that endoplasmic reticulum (ER) stress and the apoptosis it mediates play a key role in the progression of liver fibrosis, with the PERK-CHOP signaling pathway serving as a central mechanism regulating hepatic stellate cell (HSC) activation and hepatocyte damage. The present study aimed to investigate whether geniposide alleviates carbon tetrachloride (CCl_4_)-induced liver fibrosis in mice associated with reduced ER stress mediated by the protein kinase R-like ER kinase (PERK)- C/EBP homologous protein (CHOP) pathway. A mouse model of liver fibrosis was established by intraperitoneal injection of CCl_4_. Mice were randomly assigned to a Control group, a CCl_4_ group, Geniposide group, and a Pirfenidone group (positive control group). Liver function indicators and antioxidant indicators were measured, while histopathological changes and collagen deposition were evaluated by hematoxylin and eosin and Sirius Red staining. Expression of PERK-CHOP pathway-related and apoptosis-related molecules was assessed by western blotting and reverse transcription-quantitative PCR. HSC activation and hepatocyte apoptosis were determined by immunohistochemistry and terminal deoxynucleotidyl transferase dUTP nick end labeling (TUNEL) staining, respectively. The results demonstrated that geniposide significantly improved liver function, reduced oxidative stress, alleviated histopathological injury, and decreased collagen deposition and hydroxyproline content in CCl_4_-treated mice. Furthermore, geniposide significantly decreased the ratios of phosphorylated (p-) PERK/PERK and p-eukaryotic initiation factor 2α (eIF2α)/eIF2α protein expression and reduced CHOP protein levels, while downregulating the pro-apoptotic proteins Bcl-2-associated X protein and cysteine-aspartic protease 3 and upregulating the anti-apoptotic protein B-cell lymphoma 2. In addition, the number of TUNEL-positive hepatocytes exhibited a declining trend. In conclusion, geniposide alleviates CCl_4_-induced liver fibrosis, with its protective effects being associated with suppression of the PERK-CHOP signaling pathway and hepatocyte apoptosis.

## Introduction

Liver fibrosis is an excessive wound-healing response to various forms of chronic liver injury, including viral hepatitis, alcoholic liver disease and metabolic dysfunction-associated steatohepatitis. It is characterized by the excessive accumulation of extracellular matrix (ECM) within the liver, which progressively disrupts normal hepatic architecture and function (1). As fibrosis advances, it may ultimately progress to cirrhosis and hepatocellular carcinoma, posing a major global health burden (2).

Although substantial progress has been made in understanding the cellular and molecular mechanisms underlying liver fibrosis, effective therapies capable of reversing established fibrosis remain limited. Consequently, the identification of natural bioactive compounds with hepatoprotective and anti-fibrotic properties has become an important area of research.

Protein kinase RNA-like endoplasmic reticulum (ER) kinase (PERK) is one of the three principal ER stress sensors involved in the unfolded protein response (UPR) pathway (3). Upon activation, PERK undergoes autophosphorylation and subsequently phosphorylates eukaryotic initiation factor 2α (eIF2α). Phosphorylated (p-) eIF2α suppresses global protein translation while selectively promoting the translation of activating transcription factor 4 (ATF4). Under persistent or excessive ER stress, ATF4 induces the expression of C/EBP homologous protein (CHOP), a key mediator of ER stress-induced apoptosis (4,5).

CHOP promotes apoptosis by regulating the expression of apoptosis-related proteins, including downregulation of the anti-apoptotic protein B-cell lymphoma 2 (Bcl-2) and upregulation of the pro-apoptotic protein Bcl-2-associated X protein (Bax), ultimately leading to cysteine-aspartic protease (caspase) activation and cell apoptosis. Increasing evidence suggests that abnormal activation of the PERK-eIF2α-ATF4-CHOP signaling pathway plays a pivotal role in the development and progression of liver fibrosis. In addition to promoting hepatocyte apoptosis, this pathway has been implicated in hepatic stellate cell (HSC) activation and ECM deposition (6,7). As a result, modulation of the PERK-CHOP signaling pathway may represent a potential strategy for attenuating liver fibrosis.

Geniposide, a major iridoid glycoside derived from the fruit of *Gardenia jasminoides*, has been reported to possess multiple pharmacological activities, including anti-inflammatory, anti-oxidative stress, anti-apoptotic activities and hepatoprotective effects (8). Previous *in vitro* and *in vivo* studies have demonstrated that geniposide alleviates liver injury by modulating inflammatory signaling pathways and oxidative stress, thereby slowing the progression of liver fibrosis (9,10). In addition, it has been recently suggested that geniposide may regulate the expression of ER stress-related proteins (11). However, whether geniposide exerts its anti-fibrotic effects through modulation of the PERK-CHOP signaling pathway, thereby reducing hepatocyte apoptosis and HSC activation, remains unclear. To address this knowledge gap, pirfenidone, an antifibrotic agent with established clinical use in idiopathic pulmonary fibrosis and documented anti-fibrotic activity in experimental liver fibrosis, was included as a pharmacological comparator (12). Therefore, the present study aimed to investigate the protective effects of geniposide against CCl_4_-induced liver fibrosis and to explore the potential involvement of PERK-CHOP-associated ER stress and apoptosis.

## Materials and methods

### Chemicals and reagents

Geniposide was purchased from Shanghai Yuanye Biotechnology Co., Ltd. Pirfenidone, used as the positive control drug, was purchased from Beijing Innocare Technology Co., Ltd. Rapid Transfer Buffer (20X) (cat. no. WB4600; NCM Biotech); 10X TBST Buffer (cat. no. G0004-1L; Wuhan Servicebio Technology Co., Ltd.); polyvinylidene fluoride (PVDF) Membrane (cat. no. 0000378827; Immobilon-P; MilliporeSigma); PAGE Gel Fast Preparation Kit (cat. no. PG112; Yamei Biotechnology Co, Ltd.); Prestained Protein Marker VII (cat. no. MPC2409047; Wuhan Servicebio Technology Co., Ltd.); anti-Bax Rabbit mAb (cat. no. R013644; Yamei Biotechnology Co, Ltd.); Anti-Caspase3 Rabbit mAb (cat. no. R015994; Yamei Biotechnology Co, Ltd.); anti-Bcl2 Rabbit mAb (cat. no. R010733; Yamei Biotechnology Co, Ltd.); HRP-labeled goat anti-rabbit IgG (cat. no. LF102; Yamei Biotechnology Co, Ltd.); phospho-EIF2S1 (Ser1) Rabbit pAb (cat. no. 310073; Zenbio); phospho-PERK (Thr982) Rabbit pAb (cat. no. 340846; Zenbio); eIF2 alpha (D7D3) Rabbit Monoclonal Antibody (cat. no. 5324T; Cell Signaling Technology, Inc.); CHOP (D46F1) (cat. no. 5554T; Cell Signaling Technology, Inc.); PERK (PT0310R) PT™ Rabbit mAb (cat. no. YM8183; ImmunoWay Biotechnology Company); and anti-Collagen I Rabbit pAb (cat. no. GB11022-3; Wuhan Servicebio Technology Co., Ltd.).

Liver function assay kits, hydroxyproline (HYP) assay kits, hematoxylin and eosin (H&E) staining kits, Sirius Red staining kits, and terminal deoxynucleotidyl transferase dUTP nick end labeling (TUNEL) apoptosis detection kits were purchased from Shenzhen Youpin Biotechnology Co., Ltd. TRIzol reagent was obtained from Shanghai Beyotime Biotechnology Co., Ltd. Reverse transcription-quantitative polymerase chain reaction (RT-qPCR) kits were purchased from Nanjing Novizan Biotechnology Co., Ltd.

Serum biochemical indicators, including alanine aminotransferase (ALT), aspartate aminotransferase (AST), superoxide dismutase (SOD), reduced glutathione (GSH), malondialdehyde (MDA) and HYP, were measured using commercially available assay kits obtained from Hangzhou Uping Biotechnology Co., Ltd. CCl_4_ was purchased from Acmec and diluted with corn oil (1:3, v/v) before use. Horseradish peroxidase (HRP)-conjugated secondary antibodies and western blotting reagents were purchased from Yamei Biotechnology Co, Ltd. All other chemicals and reagents used in the present study were of analytical grade.

### Animal experiments

Male C57BL/6J mice (6-8 weeks old, weighing 20-22 g) were obtained from Shanghai Jihui Laboratory Animal Co., Ltd. and housed at the Experimental Animal Center of Xiamen University under specific pathogen-free (SPF) conditions with a 12 h light/dark cycle, controlled temperature (22±2˚C), and relative humidity of 50-60%, with free access to standard chow and water. All animal procedures were approved (approval no. XMULAC20120020) by the Institutional Animal Care and Use Committee of Xiamen University (Xiamen, China). Animals were monitored daily for general health, body weight, food intake, activity, and signs of distress. Humane endpoints were implemented in accordance with the approved institutional animal protocol. No animals were excluded from the study.

After one week of acclimatization, mice were randomly divided into four groups (n=6 per group): Control group, CCl_4_ group, Geniposide group, and Pirfenidone group. No formal *a priori* sample size calculation was performed; the sample size was selected based on previous studies using comparable CCl_4_-induced liver fibrosis models. A liver fibrosis model was established by intraperitoneal injection of 25% CCl_4_-corn oil. Briefly, CCl_4_ was diluted in corn oil at a volume ratio of 1:3 (v/v) and administered intraperitoneally at 0.02 ml per mouse twice weekly for 7 weeks. This corresponded to ~1 ml/kg for a 20-g mouse. The stated injection volume refers to the diluted CCl_4_-corn oil solution. Mice in the Control group received an equal volume of corn oil without CCl_4_.

Drug treatment was initiated during the third week of CCl_4_ administration and continued concurrently until the end of the experiment. Geniposide powder and pirfenidone powder were dissolved in 0.5% sodium carboxymethyl cellulose (CMC-Na) solution before use. Mice in the Geniposide group and Pirfenidone group were orally administered geniposide (100 mg/kg/day) and pirfenidone (200 mg/kg/day), respectively, by oral gavage once daily at a volume of 0.2 ml per mouse. Mice in the control and CCl_4_ model groups received an equal volume of 0.5% CMC-Na vehicle. During the treatment period, mice in the model and treatment groups continued to receive intraperitoneal injections of the CCl_4_ mixture twice weekly. Animals were monitored daily throughout the study for signs of distress, abnormal behavior, weight loss, or illness.

At the end of the experimental period, mice were deeply anesthetized with 5% isoflurane. After confirming complete loss of consciousness, they were euthanized by cervical dislocation. Death was confirmed by the following criteria: Absence of respiratory movement (thoracic excursions), heartbeat, corneal reflex, and response to toe pinch. Blood samples were collected by orbital sinus and centrifuged at 1,711 x g for 15 min at 4˚C to obtain serum for biochemical analysis of ALT and AST. Liver tissues were rapidly excised, rinsed with ice-cold phosphate-buffered saline (PBS), and divided into three portions. One portion was fixed in 4% paraformaldehyde for 24 h, embedded in paraffin, and used for H&E staining, Sirius Red staining, immunohistochemistry (IHC) and TUNEL analysis. A second portion was preserved in RNA later solution for RT-qPCR analysis. The remaining liver tissue was snap-frozen in liquid nitrogen and stored at -80˚C for western blotting and biochemical analyses. No animals or data points were excluded from the final analysis.

### Histopathological staining

Immediately after removal, liver tissue was fixed in a 4% paraformaldehyde solution for 24 h, followed by dehydration in a gradient of ethanol, clearing with xylene, and infiltration with paraffin, after which it was embedded in a paraffin block. A microtome was used to cut the paraffin block into consecutive sections 4-µm thick, which were mounted on glass slides for observation under a brightfield microscope.

Paraffin sections were sequentially dewaxed with xylene and rehydrated with a gradient of ethanol, followed by H&E staining. The staining procedure includes: Staining with hematoxylin solution for 5-10 min, rinsing with tap water, differentiation with 1% hydrochloric acid-ethanol for several seconds, treatment with a re-blue solution, followed by staining with eosin solution for 2-3 min, dehydration with graded ethanol, clearing with xylene, and mounting with neutral resin. H&E staining is used to observe the basic morphological structure of liver tissue, with a focus on evaluating hepatocyte degeneration (such as hyaline degeneration and steatosis), necrosis, inflammatory cell infiltration (including focal or diffuse infiltration), dilation of hepatic sinuses, disruption of hepatic lobule structure, and changes in the portal triad.

Paraffin-embedded liver sections were stained with Sirius Red to visualize collagen fiber deposition. After dewaxing and rehydration, the section was stained with Sirius Red solution at room temperature (20-25˚C) for 1 h, then rapidly rinsed with dilute acetic acid, dehydrated to transparency, and mounted. This staining was primarily used to assess the degree of liver fibrosis and to observe the formation and distribution of fibrous septa. Images were captured from randomly selected fields using an Olympus BX53 microscope. The collagen-positive area was quantified using ImageJ software and expressed as the collagen volume fraction (CVF), calculated as the percentage of Sirius Red-positive area relative to the total tissue area. All histological analyses were reviewed by two pathologists using a single-blind method.

### IHC

Paraffin sections were deparaffinized and rehydrated, followed by antigen retrieval using a citrate buffer (pH 6.0) in a microwave oven (heated at 95˚C for 15 min) and allowed to cool naturally to room temperature. To inactivate endogenous peroxidase, the sections were incubated with a 3% hydrogen peroxide solution at room temperature for 10 min. Non-specific binding sites were then blocked with 3% BSA (cat. no. GC305010; Wuhan Servicebio Technology Co., Ltd.) and incubated at room temperature (20-25˚C) for 30 min. After discarding the serum, rabbit anti-α-smooth muscle actin (α-SMA; 1:200; cat. no. GB121364; Wuhan Servicebio Technology Co., Ltd.) and anti-collagen I (1:200; cat. no. GB11022-3; Wuhan Servicebio Technology Co., Ltd.) primary antibodies were added dropwise and incubated overnight at 4˚C in a humid chamber. The next day, after washing with PBS, HRP-conjugated goat anti-rabbit secondary antibody (1:1,000; cat. no. LF102; Yamei Biotechnology Co, Ltd.) was added and incubated at room temperature for 1 h. After thorough washing with PBS, 3,3'-diaminobenzidine (DAB) was used as the chromogenic substrate, and the samples were developed for 5-10 min at room temperature in the dark. The intensity of the staining was monitored under a microscope, and the reaction was terminated with distilled water. The cell nuclei were counterstained with hematoxylin. Images were captured using an Olympus BX53 microscope equipped with a DP26 digital camera. The positive staining area was quantified using ImageJ software.

For TUNEL analysis, antigen retrieval was performed on the treated paraffin sections by incubating them with 20 µg/ml proteinase K at 37˚C for 20 min. Following washing with PBS, the sections were incubated with the TUNEL reaction mixture in a dark, humid chamber at 37˚C for 1 h, according to the instructions of the TUNEL apoptosis detection kit. After thorough rinsing with PBS, the cell nuclei were counterstained with 4',6-diamino-2-phenylindole (DAPI, 1 µg/ml) for 5 min, and the slides were mounted with an anti-fade mounting medium. Images were observed and acquired using a Nikon fluorescence microscope (Nikon Corporation). The percentage of TUNEL-positive cells (red fluorescence) relative to the total number of DAPI-positive cells, the apoptosis index, was calculated to reflect the level of hepatocyte apoptosis. All image analyses were performed under uniform exposure and parameter settings to ensure the comparability of results.

### Biochemical analysis

After blood samples were collected, they were left to stand at room temperature for 30 min to allow natural clotting. The samples were then centrifuged at 1,711 x g for 15 min to separate the serum, which was aliquoted and stored at -80˚C for future use. Using the rate method, a fully automated biochemical analyzer and a commercial assay kit were employed, strictly following the manufacturer's instructions, to measure serum ALT and AST activity, expressed in U/l, to assess the extent of hepatocyte damage.

After removing the liver tissue, an appropriate amount (~0.1 g) was weighed and mixed with pre-chilled PBS (pH 7.4) at a 1:9 (w/v) ratio. A 10% homogenate was prepared using a tissue homogenizer on an ice bath. The mixture was then centrifuged at 4˚C and 1,711 x g for 10 min, and the supernatant was collected for biochemical analysis. SOD activity was determined using the WST-1 method at a detection wavelength of 450 nm, with results expressed in U/mg protein; GSH content was determined using the DTNB colorimetric method at a detection wavelength of 412 nm, with results expressed in µmol/g protein; MDA content was measured using the thiobarbituric acid method at a wavelength of 532 nm, with results expressed in nmol/mg protein; HYP content was measured using the alkaline hydrolysis method at a wavelength of 550 nm, with results expressed in mg/mg tissue. These biochemical parameters were used to assess the effects of geniposide on liver injury, oxidative stress, and hepatic fibrosis in CCl_4_-treated mice.

### Western blot analysis

Total protein was extracted from liver tissues using RIPA lysis buffer (cat. no. R0010; Beijing Solarbio Science & Technology Co., Ltd.), and protein concentrations were determined using a BCA protein assay kit. Equal amounts of protein (30 µg per lane) were separated by 10% SDS-PAGE and transferred onto PVDF membranes. After blocking with 5% non-fat milk for 1 h at room temperature, the membranes were incubated overnight at 4˚C with primary antibodies against p-PERK, PERK, p-eIF2α, eIF2α, CHOP, α-SMA, Bax, Bcl-2, Caspase-3 and β-actin. The membranes were then incubated with HRP-conjugated secondary antibodies (1:5,000; cat. no. LF102; Yamei Biotechnology Co, Ltd.) for 1 h at room temperature. Protein bands were visualized using an enhanced chemiluminescence (ECL) detection kit and quantified using ImageJ software (version 1.x, National Institutes of Health).

### RT-qPCR

Liver tissues stored at -80˚C were used for total RNA extraction with TRIzol reagent according to the manufacturer's instructions. The concentration and purity of the extracted RNA were determined using an ultra-micro spectrophotometer. Subsequently, 1 µg of total RNA was reverse transcribed into complementary DNA (cDNA) using a reverse transcription kit (cat. no. R323-01; Vazyme Biotech Co., Ltd.) according to the manufacturer's instructions. RT-qPCR was performed using SYBR Green Master Mix with cDNA as the template to determine the relative mRNA expression levels of PERK, eIF2α, CHOP, Caspase-3, Bcl-2 and Bax with β-actin serving as the internal reference gene. Primer sequences were designed based on the corresponding gene sequences obtained from GenBank (https://www.ncbi.nlm.nih.gov/genbank/). PCR amplification was performed according to the manufacturer's instructions and melting curve analysis was conducted to confirm the specificity of the amplified products. PCR amplification was performed with the following thermal cycling conditions: initial denaturation at 95˚C for 30 sec, followed by 40 cycles of denaturation at 95˚C for 10 sec, annealing at 60˚C for 30 sec, and extension at 72˚C for 30 sec. Melting curve analysis was conducted to confirm the specificity of the amplified products. Each sample was analyzed in triplicate, and the relative mRNA expression levels were calculated using the 2^-ΔΔCq^ method (13). The primer sequences used in the present study are listed in Table I.

### Statistical analysis

Data are presented as the mean ± SD. Normality was assessed using the Shapiro-Wilk test. Comparisons among multiple groups were performed using one-way ANOVA followed by Tukey's multiple-comparisons test. Body-weight changes over time were analyzed using two-way repeated-measures ANOVA, with groups and time as factors, followed by an appropriate multiple-comparisons test. All replicates in the experiments were biological replicates. A two-sided P<0.05 was considered to indicate a statistically significant difference. Statistical analyses were performed using GraphPad Prism 10 (Dotmatics).

## Results

### Effects of Geniposide on the general condition and liver function of mice with liver fibrosis

During the experiment, mice in the Control group exhibited normal behavioral patterns, healthy physical appearances, and steady weight gain. By contrast, mice in the CCl_4_ group displayed lethargy, reduced physical activity, stunted or decreased weight gain, and a significantly elevated liver-to-body weight ratio. Compared with the CCl_4_ group, the general condition of mice in the Geniposide group and the Pirfenidone group was markedly improved. Biochemical analysis demonstrated that serum ALT and AST levels were significantly elevated in the CCl_4_ group compared with the Control group (P<0.001; P<0.05). Levels of HYP and MDA in liver tissue increased (P<0.001), while levels of GSH and SOD decreased (P<0.001). Following treatment with geniposide, all of these parameters showed a certain degree of recovery compared with the CCl_4_ group (P<0.001, P<0.01 or P<0.05). These results indicate that geniposide can effectively alleviate the severity of inflammation, the extent of collagen deposition, and oxidative stress in liver fibrosis in mice (Fig. 1A-H).

### Effects of geniposide on liver histopathology

H&E staining showed that a large number of hepatocytes in the CCl_4_ group exhibited ballooning degeneration, fatty degeneration and focal necrosis. Prominent inflammatory cell infiltration was observed within both the portal areas and hepatic lobules, accompanied by the formation of noticeable fibrous septa. Following geniposide treatment, hepatocyte damage was markedly attenuated, inflammatory infiltration subsided, and the fibrous septa became thinner and fragmented, indicating a clear trend toward tissue structural recovery. Sirius Red staining showed extensive collagen deposition in the portal areas and around the central veins of the CCl_4_ group, resulting in the formation of broad fibrous septa. Quantitative analysis showed that geniposide treatment significantly reduced the CVF (P<0.05), reflecting a significant reduction in ECM deposition, which was consistent with the results of HYP content measurement. In addition, IHC analysis showed that the expression of α-SMA and collagen I was significantly reduced following geniposide treatment. In addition, western blot analysis confirmed the reduction in α-SMA expression (Fig. 2A-D).

### Effects of geniposide on the PERK-CHOP signaling pathway

Western blot analysis showed that the ratios of p-PERK/PERK and p-eIF2α/eIF2α, as well as the expression level of CHOP, were significantly increased in the CCl_4_ group compared with the control group (P<0.05; P<0.01; P<0.001), indicating activation of the PERK-CHOP signaling pathway. Geniposide treatment significantly reduced the p-PERK/PERK and p-eIF2α/eIF2α ratios and CHOP expression compared with the model group (P<0.05; P<0.001). These findings suggest that the protective effects of geniposide were associated with reduced CCl_4_-induced ER stress and PERK-CHOP-associated signaling (Fig. 3A-H).

### Effects of geniposide on hepatic cell apoptosis

TUNEL staining results showed that a large number of TUNEL-positive cells were observed in the liver tissue of the CCl_4_ group, primarily distributed around the fibrous septa and in areas of severely damaged liver parenchyma; the apoptosis index was significantly higher than that of the Control group (P<0.001). Following treatment with geniposide, the number of apoptotic cells decreased markedly, and the apoptosis index also decreased significantly (Fig. 4A and B). Consistent with the TUNEL results, western blot analysis showed that in the CCl_4_ group, the expression of the pro-apoptotic proteins Bax and caspase-3 in liver tissue was significantly increased (P<0.05; P<0.001); although the expression of the anti-apoptotic protein Bcl-2 was also increased (P<0.05), the Bax/Bcl-2 ratio was still elevated compared with the normal group (P<0.05), suggesting aggravated hepatocyte apoptosis (Fig. 4C-G). After treatment with geniposide, all of these indicators improved. The CHOP pathway suppresses the expression of the anti-apoptotic protein Bcl-2 and triggers the translocation of Bax from the cytoplasm to the outer mitochondrial membrane, where it aggregates and forms pores, leading to the release of apoptotic factors such as cytochrome C and initiating the apoptotic program. When the Bax/Bcl-2 ratio is high, cells tend to survive; when the ratio is low, they tend to undergo apoptosis. These results suggested that PERK-CHOP-associated apoptotic signaling may be related to the degree of liver fibrosis (Fig. 4).

### Effects of geniposide on the mRNA expression of PERK-CHOP pathway- and apoptosis-related genes

RT-qPCR analysis showed that, compared with the Control group, the CCl_4_ group had significantly higher mRNA levels of PERK, eIF2α and CHOP (P<0.01); and the mRNA levels of apoptosis-related genes Caspase-3 and Bax were also significantly increased (P<0.01), while the mRNA level of the anti-apoptotic gene Bcl-2 decreased (P<0.05). Compared with the CCl_4_ group, the Geniposide group significantly reduced the mRNA levels of PERK, eIF2α, CHOP, Caspase-3 and Bax (P<0.05 or P<0.01), and increased the mRNA level of Bcl-2 (P<0.05). These findings further support an association between geniposide treatment and reduced expression of PERK-CHOP pathway-related genes at the transcriptional level (Fig. 5A-F).

## Discussion

*Establishment of a liver fibrosis model and evaluation of the therapeutic effect of geniposide*. CCl_4_ can cause chemical damage, which gets amplified through inflammation and eventually leads to liver fibrosis in mice through ECM deposition mediated by HSCs. This process involves oxidative stress, ER stress, multiple signaling pathways and immune cell regulation (14). In the present study, mice in the CCl_4_ group showed significant liver function impairment, marked histopathological changes in the liver, and extensive collagen deposition, indicating successful establishment of the liver fibrosis model. Following geniposide treatment, serum liver function parameters were markedly improved, hepatic pathological damage was alleviated, and both CVF and hydroxyproline content were significantly reduced. These findings suggest that geniposide exerts a protective effect against CCl_4_-induced liver fibrosis in mice.

Given its reported anti-inflammatory, antioxidant and anti-apoptotic activities, geniposide may exert broader biological effects than those reflected by the PERK-CHOP-associated mechanism investigated in the present study. However, direct comparisons of efficacy, safety, pharmacokinetics and bioavailability between geniposide and established antifibrotic agents require further investigation.

### Regulatory effects of geniposide on the PERK-CHOP signaling pathway

ER stress has been recognized as an important contributor to the initiation and progression of liver fibrosis. Glucose-regulated protein 78 (GRP78), a key ER molecular chaperone, is commonly regarded as a marker of ER stress activation (15). Under persistent ER stress conditions, activation of the PERK signaling pathway promotes phosphorylation of eIF2α, subsequently enhancing ATF4 and CHOP expression and ultimately triggering apoptosis-related signaling cascades. As a central transcription factor in this pathway, CHOP regulates the expression of apoptosis-related proteins, including the upregulation of Bax, thereby promoting activation of the apoptotic cascade (4,16). In the present study, the protein expression levels of p-PERK, p-eIF2α and CHOP were significantly increased in the model group, suggesting activation of PERK-CHOP-associated signaling. Following geniposide treatment, the expression levels of these proteins were significantly decreased compared with those in the CCl_4_ model group (P<0.05 or P<0.01), suggesting that geniposide treatment was associated with reduced ER stress and PERK-CHOP signaling. RT-qPCR analysis further supported these findings, showing that geniposide significantly reduced the mRNA expression levels of PERK, eIF2α and CHOP, suggesting that its effects may occur, at least in part, at the transcriptional level.

### Geniposide reduces PERK-CHOP pathway-associated apoptosis

The present study found that geniposide significantly reduced MDA levels while increasing SOD and GSH, suggesting that its antioxidant capacity may play an upstream role in its anti-fibrotic effects. Oxidative stress is a well-established trigger of ER stress; reactive oxygen species can disrupt the redox balance within the ER, promote the accumulation of misfolded proteins, and consequently activate UPR sensors such as PERK (17). Therefore, a plausible interpretation is that geniposide first attenuates oxidative damage induced by CCl_4_ metabolism through its intrinsic antioxidant activity, which in turn reduces the initiation of ER stress and ultimately leads to a passive downregulation of PERK-CHOP pathway activity.

CHOP is considered one of the most important effector molecules in the ER stress-induced apoptosis pathway (4). CHOP activates the mitochondrial apoptosis pathway by promoting Bax translocation to the mitochondrial membrane, ultimately leading to the cleavage and activation of Caspase-3 and apoptosis (18,19). In the present study, TUNEL staining and analysis of Bax, Bcl-2 and Caspase-3 showed a large number of apoptotic cells in the CCl_4_ group, and geniposide intervention significantly reduced hepatocyte apoptosis. It should be noted that the biological effects of ER stress may differ among hepatic cell types. Hepatocyte apoptosis directly contributes to liver parenchymal injury and subsequent repair responses, whereas activated HSCs exhibit relatively strong resistance to apoptosis and may instead be regulated through non-apoptotic ER stress-related mechanisms (20,21). Therefore, the reduction effect of geniposide on CHOP-associated signaling may not only alleviate hepatocyte apoptosis while the concurrent reduction in HSC activation may further contribute to the anti-fibrotic effects of geniposide.

### Innovation and limitations of the study

The present study systematically investigated the protective effects of geniposide against liver fibrosis and explored its underlying molecular mechanism from the perspective of ER stress. The findings suggest that the anti-fibrotic effects of geniposide are associated with reduced ER stress-related apoptotic signaling and decreased activation of the PERK-CHOP pathway. Geniposide can also reduce liver cell apoptosis associated with reduced the protein expression of Bax and Caspase-3 and promoting the protein expression of Bcl-2, which further supports its anti-apoptotic effects. These findings provide experimental evidence supporting the potential of geniposide as a candidate for further investigation in liver fibrosis.

However, the present study also has certain limitations. The present study mainly focused on *in vivo* pharmacological effects and pathway-related protein expression, while lacking direct mechanistic evidence from primary HSCs or HSC cell lines. Therefore, the direct regulatory effect of geniposide on HSC activation through the PERK-CHOP pathway requires further investigation. Thus, the direct binding relationship between geniposide and PERK remains unclear. GRP78/BiP, a commonly used marker of ER stress, was not assessed in the present study. Future studies should include GRP78/BiP and other upstream ER stress markers to further characterize the involvement of ER stress and determine whether geniposide affects ER stress upstream of PERK. Moreover, the present study did not include pathway-specific inhibition, gene knockdown/knockout, or rescue experiments; therefore, a direct causal role of PERK-CHOP signaling in the anti-fibrotic effects of geniposide cannot be established. Future studies could employ hepatocyte or HSC-specific gene knockout models, as well as gene silencing approaches in HSC-T6 or other hepatic cell lines, to further clarify the specific role of the PERK-CHOP pathway in the antifibrotic effects of geniposide. In addition, further investigation into the pharmacokinetic properties and clinical translational potential of geniposide is warranted. A schematic illustration of the proposed mechanism is presented in Fig. S1.

### Conclusions and prospects

The present study demonstrated that geniposide can effectively improve CCL_4_-induced liver fibrosis in mice. Its protective effects were associated with reduced ER stress-related PERK-CHOP signaling, decreased hepatocyte apoptosis, and inhibition of HSC activation. These findings provide experimental evidence for the potential anti-fibrotic effects of geniposide and suggest that PERK-CHOP signaling may be involved in its protective actions. Further studies using pathway-specific genetic or pharmacological approaches are needed to establish the direct role of PERK-CHOP signaling in the anti-fibrotic effects of geniposide.

## Supplementary Material

Proposed mechanism potentially underlying the protective effects of geniposide against CCl_4_-induced liver fibrosis. Geniposide is associated with reduced PERK-CHOP-related ER stress and apoptosis, accompanied by decreased Bax and cleaved Caspase-3 expression, increased Bcl-2 expression, reduced hepatocyte apoptosis, and inhibition of HSC activation. CCl_4_, carbon tetrachloride; PERK, protein kinase R-like ER kinase; CHOP, C/EBP homologous protein; ER, endoplasmic reticulum; Bax, Bcl-2-associated X protein; Bcl-2, B-cell lymphoma 2; ALT, alanine aminotransferase; AST, aspartate aminotransferase; eIF2α, eukaryotic initiation factor 2α; ATF4, activating transcription factor 4; CHOP, C/EBP homologous protein; HSC, hepatic stellate cell; p-, phosphorylated; GRP78, glucose-regulated protein 78; Caspase-3, cysteine aspartic protease 3.

## Figures and Tables

**Figure 1:**
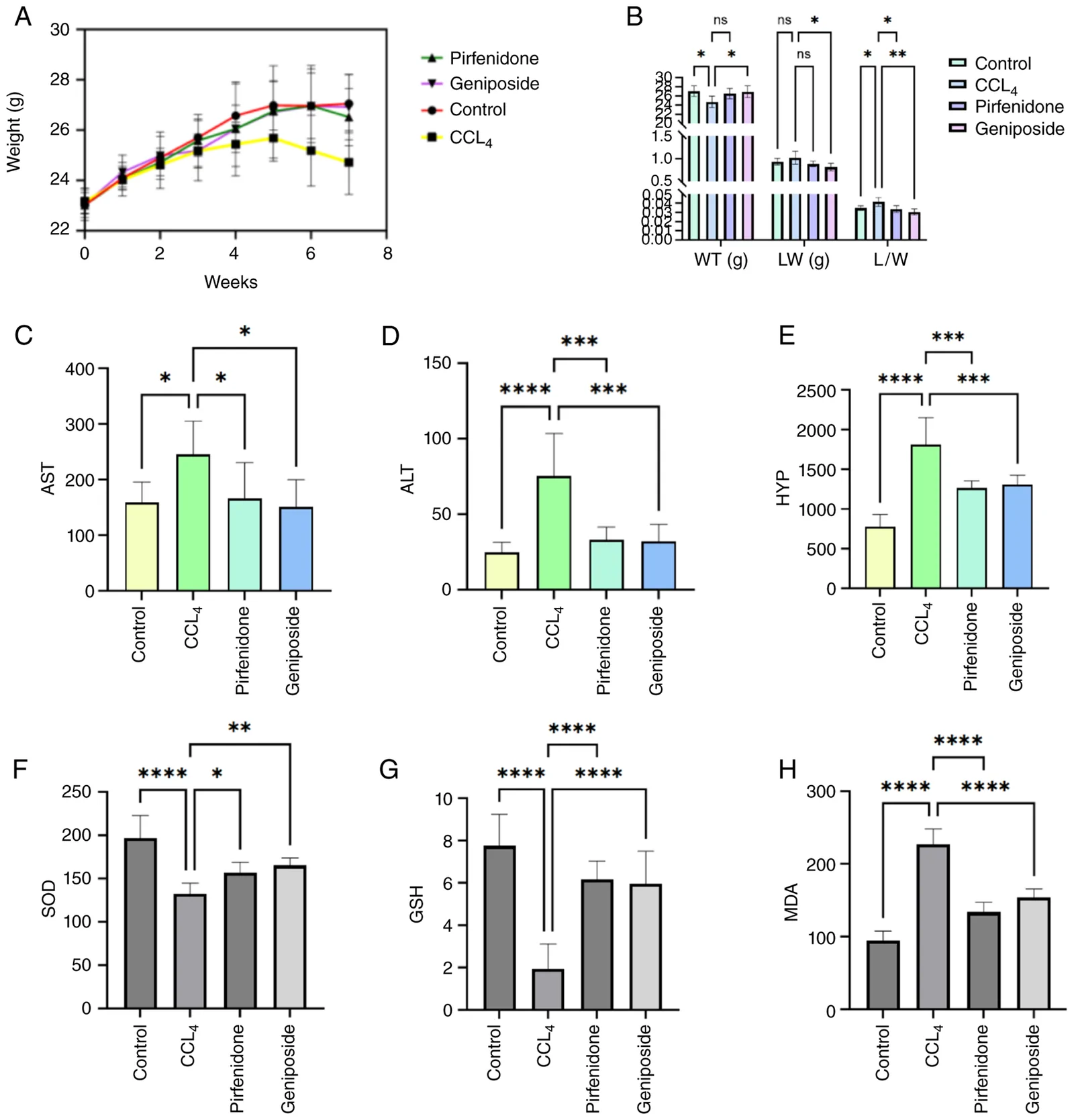
Effects of geniposide on general condition, liver function, and oxidative stress in CCl_4_-induced liver fibrosis mice. (A) Body weight change curve: Changes in body weight (g) of mice in each group throughout the experimental period (7 weeks). (B) Body weight and liver weight: the left panel shows the final body weight (WT, g), the middle panel shows the liver weight at sacrifice (LW, g), and the right panel shows the liver wet weight to body weight ratio (L/W). (C-E) Serum AST and ALT levels and HYP content in each group. (F-H) Hepatic SOD, GSH and MDA levels in each group. All data are expressed as the mean ± SD. Statistical analysis was performed using one-way ANOVA. ^*^P<0.05, ^**^P<0.01, ^***^P<0.001 and ^****^P<0.0001 vs. the CCl_4_ group; (n=6). CCl_4_, carbon tetrachloride; ALT, alanine aminotransferase; AST, aspartate aminotransferase; HYP, hepatic hydroxyproline; SOD, superoxide dismutase; GSH, glutathione; MDA, malondialdehyde; ns, not significant.

**Figure 2:**
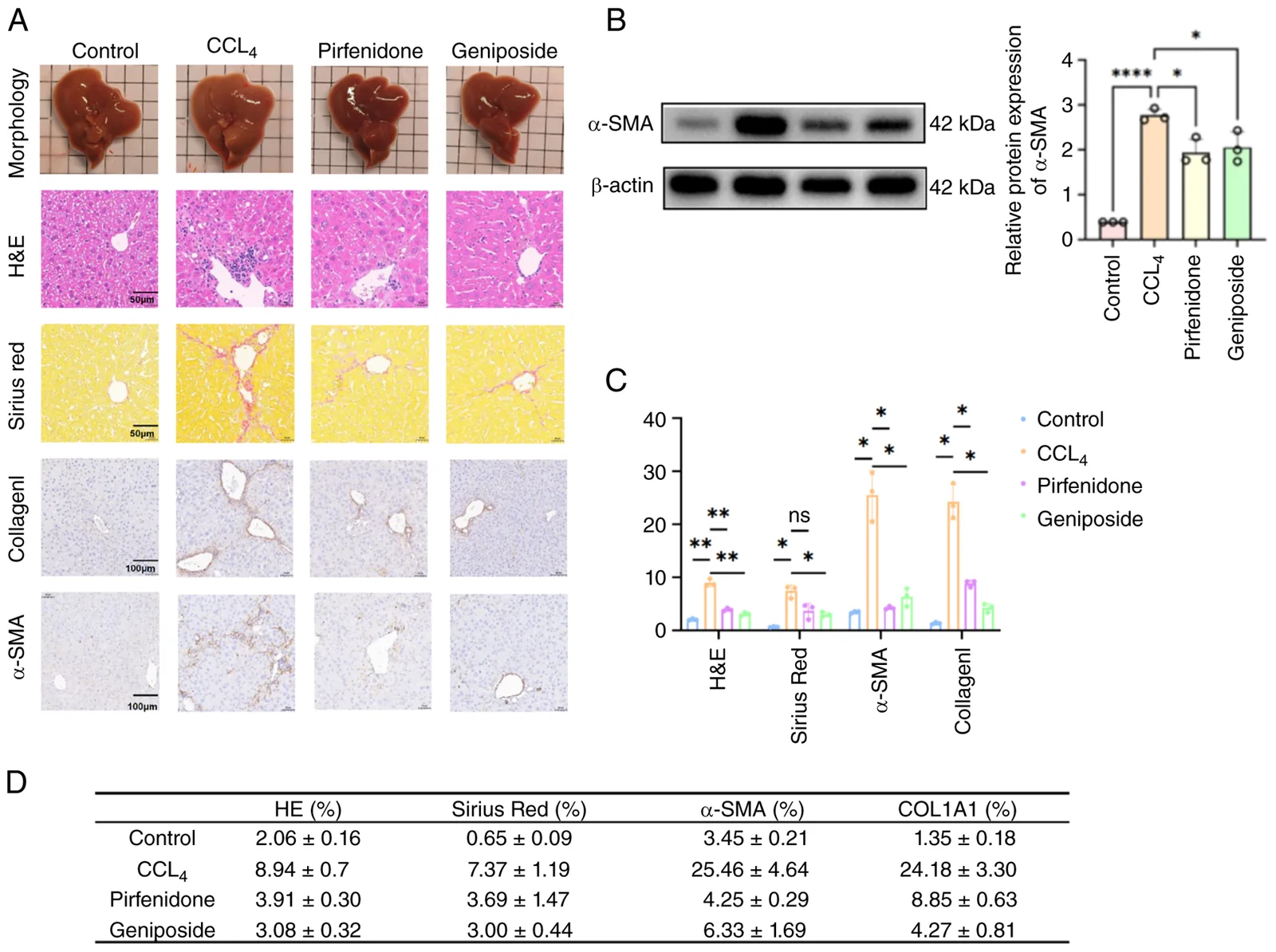
Effects of geniposide on liver histopathological changes and fibrosis in CCl_4_-induced liver fibrosis mice. (A) Representative gross morphology of liver tissues, H&E staining, Sirius Red staining, and IHC staining of α-SMA and Collagen I in each group. (B) Representative western blot bands and quantitative analysis of α-SMA protein expression in liver tissues. (C) Quantitative analysis of H&E-positive injury area, Sirius Red-positive fibrotic area, and α-SMA- and Collagen I-positive areas in each group. (D) Statistical summary of histopathological and IHC quantification results. Data are presented as the mean ± SD. Statistical analysis was performed using one-way ANOVA. ^*^P<0.05, ^**^P<0.01 and ^****^P<0.0001 vs. the CCl_4_ group; (n=3; magnification, x200). CCl_4_, carbon tetrachloride; α-SMA, alpha-smooth muscle actin; IHC, immunohistochemical; ns, not significant.

**Figure 3:**
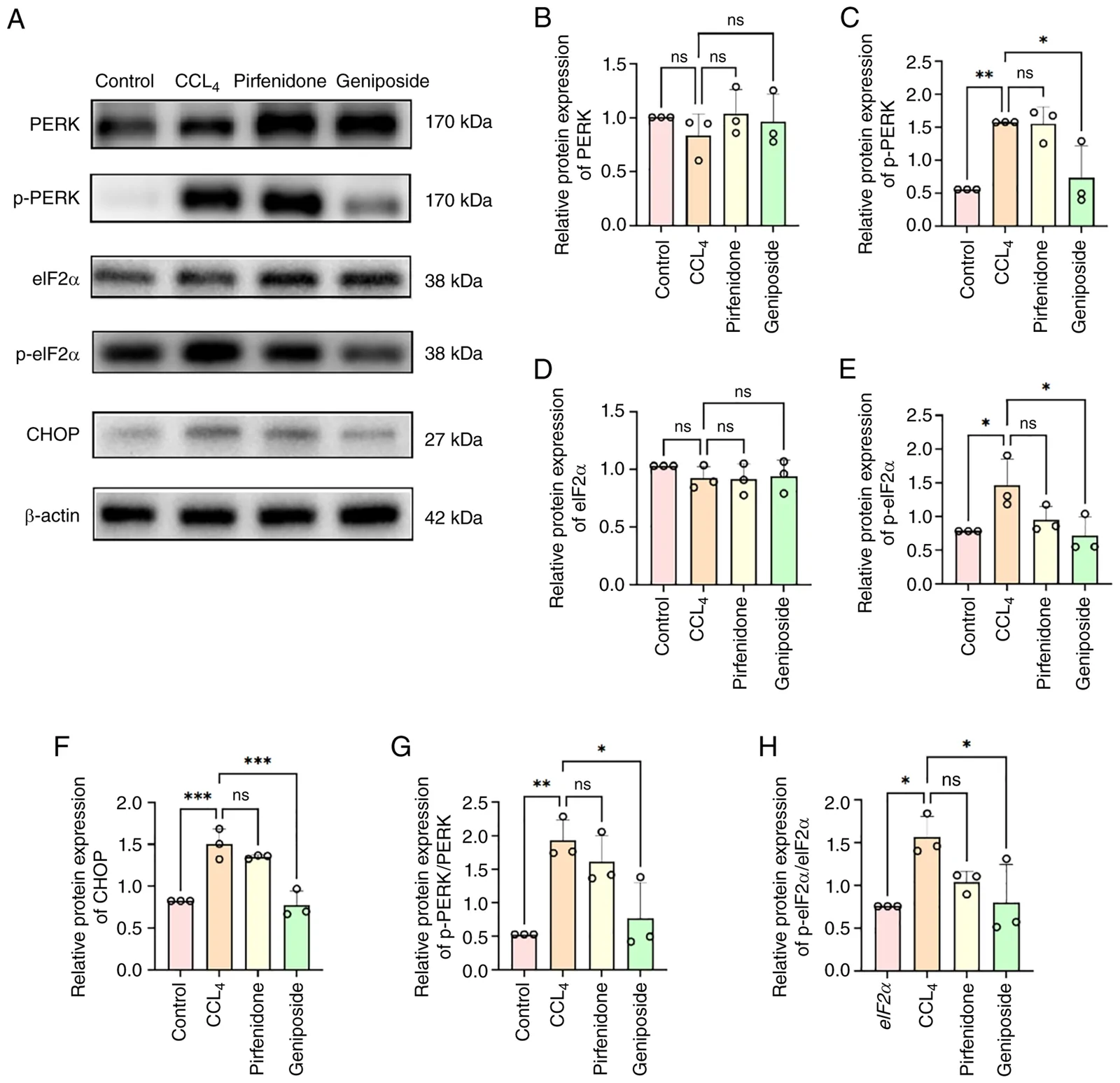
Western blot analysis of PERK/eIF2α/CHOP signaling pathway-related proteins in liver tissues of CCl_4_-induced liver fibrosis mice. (A) Representative Western blot bands of p-PERK, p-eIF2α, PERK, eIF2α and CHOP protein expression in liver tissues from each group. (B-H) Densitometric analysis of (B) PERK, (C) p-PERK, (D) eIF2α, (E) p-eIF2α, (F) CHOP, (G) p-PERK/PERK and (H) p-eIF2α/eIF2α protein expression levels in each group. Data are presented as the mean ± SD. Statistical analysis was performed using one-way ANOVA. ^*^P<0.05, ^**^P<0.01 and ^***^P<0.001 vs. the CCl_4_ group; (n=3). PERK, protein kinase R-like ER kinase; eIF2α, eukaryotic initiation factor 2α; CHOP, C/EBP homologous protein; CCl_4_, carbon tetrachloride; p-, phosphorylated; ns, not significant.

**Figure 4:**
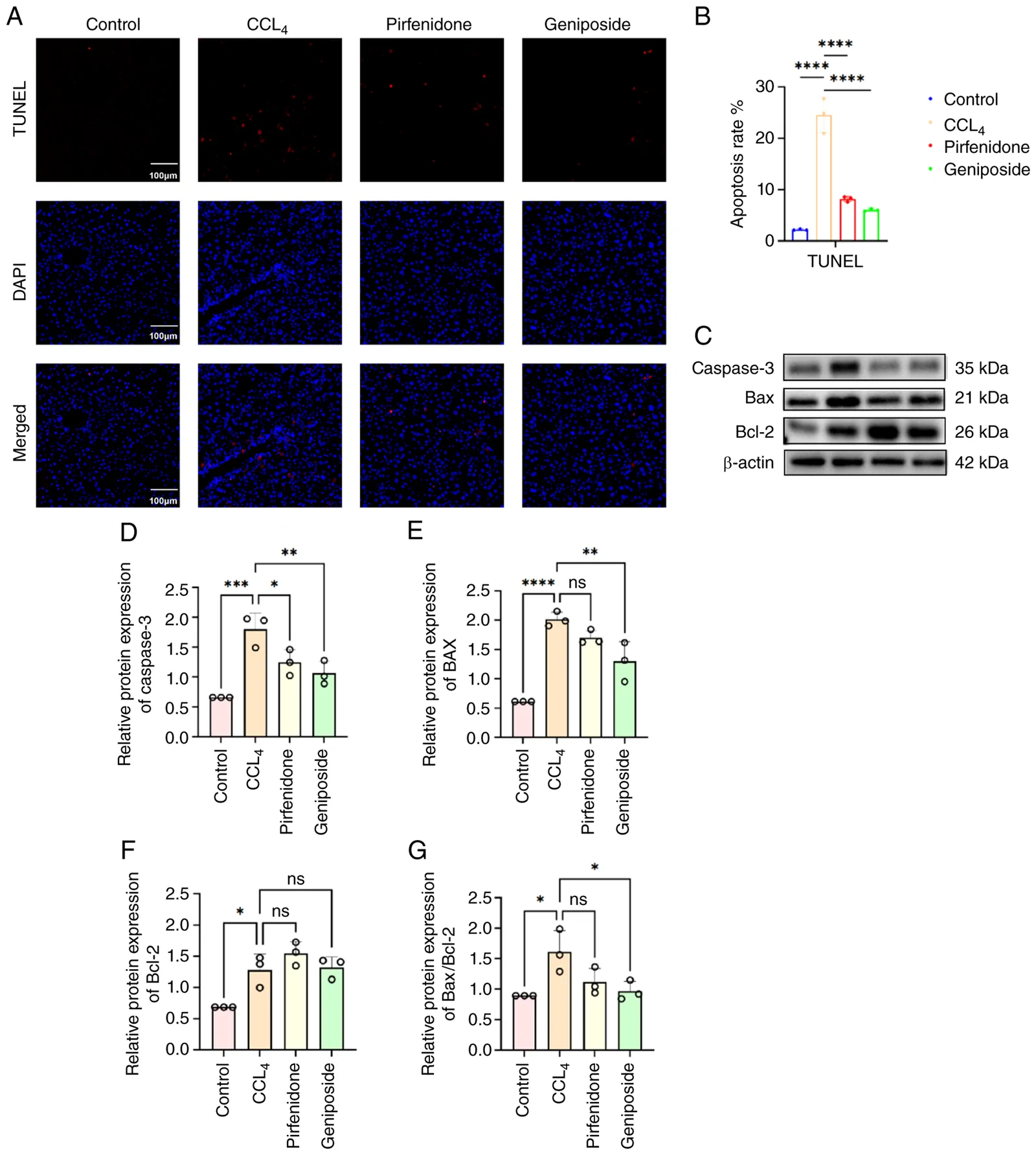
Effects of geniposide on hepatocyte apoptosis in CCl_4_-induced liver fibrosis mice. (A) Representative TUNEL staining images of liver tissue sections from each group. TUNEL-positive cells are shown in red, and nuclei are counterstained with DAPI (blue). (B) Quantitative analysis of the apoptosis rate in liver tissues from each group. (C) Representative western blot bands of cleaved Caspase-3, Bax, Bcl-2 and Bax/Bcl-2 protein expression in liver tissues from each group. (D-G) Densitometric analysis of (D) Caspase-3, (E) Bax, (F) Bcl-2 and (G) Bax/Bcl-2. Data are presented as the mean ± SD (n=3). Statistical analysis was performed using one-way ANOVA. ^*^P<0.05, ^**^P<0.01, ^***^P<0.001 and ^****^P<0.0001 vs. the CCl_4_ group; (n=3; magnification, x400). CCl_4_, carbon tetrachloride; TUNEL, terminal deoxynucleotidyl transferase dUTP nick end labeling; Bax, Bcl-2-associated X protein; Bcl-2, B-cell lymphoma 2; caspase-3, cysteine-aspartic protease 3; Bax, Bcl-2-associated X protein; ns, not significant.

**Figure 5:**
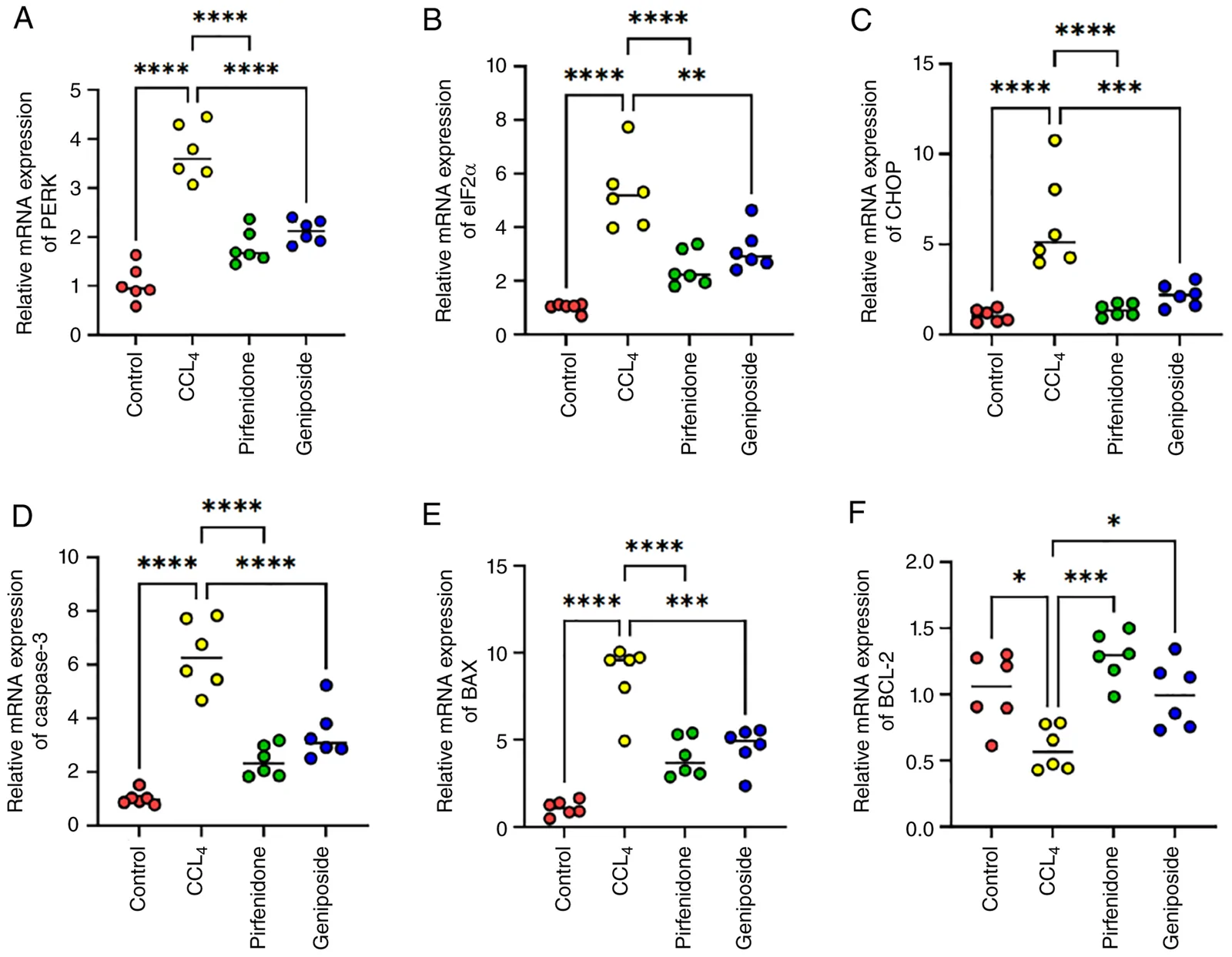
Relative mRNA expression levels of PERK, eIF2α, CHOP, Caspase-3, Bax and Bcl-2 in liver tissues detected by reverse transcription-quantitative PCR. (A-F) Relative mRNA expression levels of (A) PERK, (B) eIF2α, (C) CHOP, (D) Caspase-3, (E) Bax and (F) Bcl-2 in liver tissues from each group. Data are presented as the mean ± SD. Statistical analysis was performed using one-way ANOVA. ^*^P<0.05, ^**^P<0.01, ^***^P<0.001 and ^****^P<0.0001 vs. the CCl_4_ group; (n=6). PERK, protein kinase R-like ER kinase; eIF2α, eukaryotic initiation factor 2α; CHOP, C/EBP homologous protein; caspase-3, cysteine-aspartic protease 3; Bax, Bcl-2-associated X protein; Bcl-2, B-cell lymphoma 2.

**Table I:** Primer sequences used for reverse transcription-quantitative PCR analysis.

Gene name	Primer sequence (5'-3')
PERK	F: GACGAATCGCTGCACTGGAT
	R: AAGTTTTGTGGGTGCCCTCTG
eIF2α	F: CCTGGAGCAATGGAGAAAAAGTA
	R: AGTAGTGTAAGGCTGCCACG
CHOP	F: TCTTGAGCCTAACACGTCGATT
	R: ACGTGGACCAGGTTCTCTCT
Caspase-3	F: GCTTGGAACGGTACGCTAAG
	R: CCACTGACTTGCTCCCATGT
Bax	F: AAACTGGTGCTCAAGGCCC
	R: GGTCCCGAAGTAGGAGAGGA
Bcl-2	F: CACCCCTGGTGGACAACATC
	R: TATAGTTCCACAAAGGCATCCCAG
β-actin	F: GGCGGACTGTTACTGAGCTG
	R: CTGCGCAAGTTAGGTTTTGTCA

PERK, protein kinase R-like ER kinase; eIF2α, eukaryotic initiation factor 2α; CHOP, C/EBP homologous protein; Caspase-3, cysteine-aspartic protease 3; Bax, Bcl-2-associated X protein; Bcl-2, B-cell lymphoma 2; F, forward; R, reverse.

## Data Availability

The data generated in the present study are included in the figures and/or tables of this article.
