# Supplementary material for: Geniposide alleviates CCl4-induced liver fibrosis: Potential involvement of the PERK-CHOP signaling pathway
Source: Biomed Rep. 2026 Sep 30;25(5):136. doi: 10.3892/br.2026.2209 (PMC13688171; doi:10.3892/br.2026.2209)

Figure S1. Proposed mechanism potentially underlying the protective effects of geniposide against CCl_4-induced liver fibrosis. Geniposide is associated with reduced PERK-CHOP-related ER stress and apoptosis, accompanied by decreased Bax and cleaved Caspase-3 expression, increased Bcl-2 expression, reduced hepatocyte apoptosis, and inhibition of HSC activation. CCl_4, carbon tetrachloride; PERK, protein kinase R-like ER kinase; CHOP, C/EBP homologous protein; ER, endoplasmic reticulum; Bax, Bcl-2-associated X protein; Bcl-2, B-cell lymphoma 2; ALT, alanine aminotransferase; AST, aspartate aminotransferase; eIF2α, eukaryotic initiation factor 2α; ATF4, activating transcription factor 4; CHOP, C/EBP homologous protein; HSC, hepatic stellate cell; p-, phosphorylated; GRP78, glucose-regulated protein 78; Caspase-3, cysteine-aspartic protease 3.

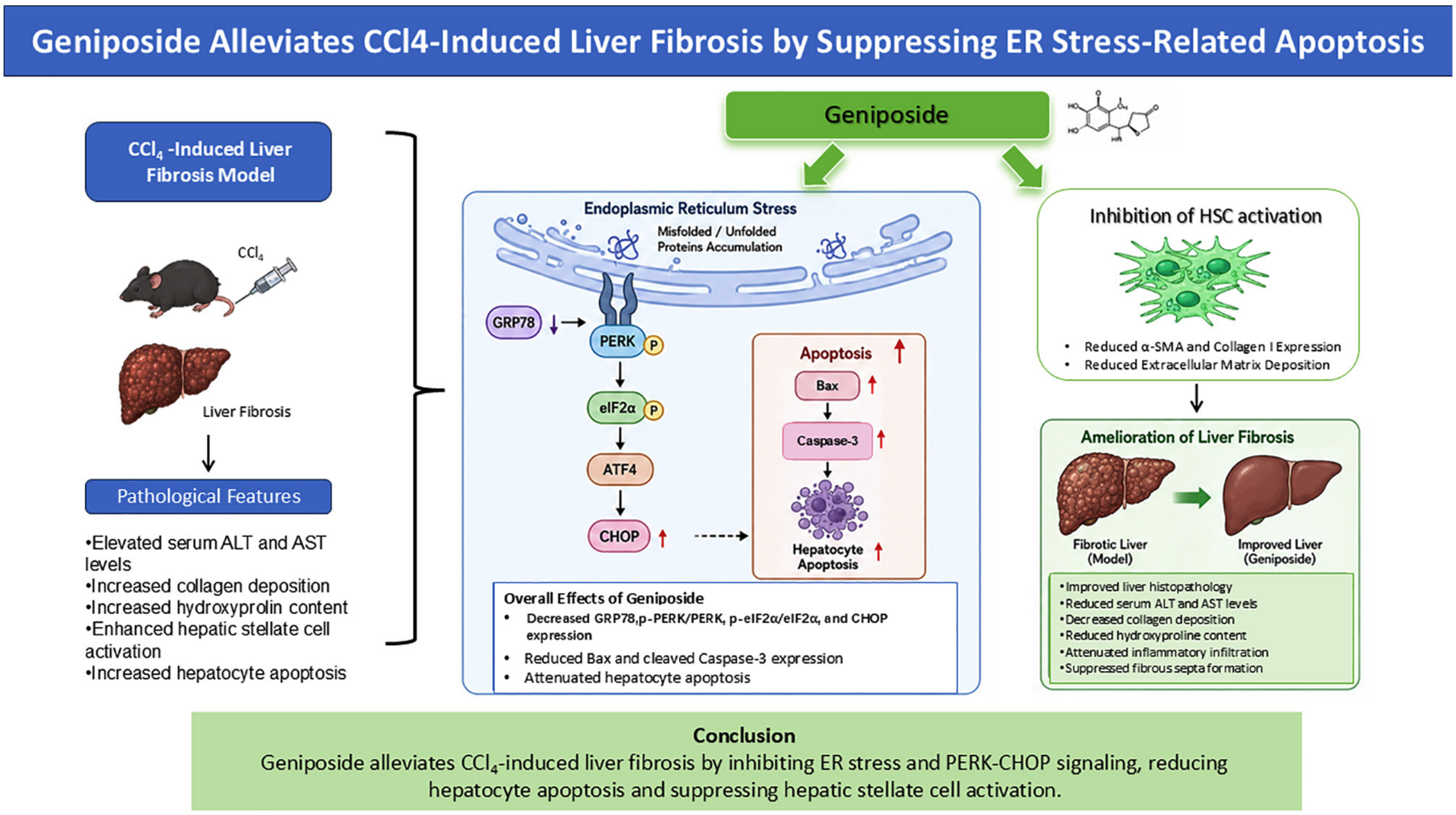

Supplement: Supplementary file 1 [file Supplementary_Data.pdf]
